# Evidence of Different IL-1β Activation Pathways in Innate Immune Cells From Indeterminate and Cardiac Patients With Chronic Chagas Disease

**DOI:** 10.3389/fimmu.2019.00800

**Published:** 2019-04-18

**Authors:** Nayara I. Medeiros, Bruna F. Pinto, Silvana M. Elói-Santos, Andréa Teixeira-Carvalho, Luísa M. D. Magalhães, Walderez O. Dutra, Rodrigo Correa-Oliveira, Juliana A. S. Gomes

**Affiliations:** ^1^Imunologia Celular e Molecular, Instituto René Rachou, FIOCRUZ, Belo Horizonte, Brazil; ^2^Laboratório de Biologia das Interações Celulares, Departamento de Morfologia, Universidade Federal de Minas Gerais, Belo Horizonte, Brazil; ^3^Grupo Integrado de Pesquisas em Biomarcadores, Instituto René Rachou, FIOCRUZ, Belo Horizonte, Brazil; ^4^Departamento de Propedêutica Complementar, Faculdade de Medicina, Universidade Federal de Minas Gerais, Belo Horizonte, Brazil; ^5^Inflammatory Cell Dynamics Section, Center for Cancer Research, National Institute of Health, NIH, Bethesda, MD, United States; ^6^Instituto Nacional de Ciência e Tecnologia em Doenças Topicais - INCT-DT, Belo Horizonte, Brazil

**Keywords:** IL-1β activation pathways, caspase-1-independent, gelatinases, Chagas disease, chagasic cardiomyopathy

## Abstract

**Background:** Chagas cardiomyopathy is the main fibrosing myocarditis among known heart diseases. Development of cardiomyopathy has been related to extracellular matrix (ECM) remodeling, which are controlled by matrix metalloproteinases (MMPs) and cytokines, especially interleukin (IL)-1β. The convertion of 31KDa inactive precursor, the proIL-1β in 17KDa active IL-1β peptide, is controlled by caspase-1-dependent pathway, associated with inflammasomes. Other caspase-1 independent mechanisms mediated by proteases, especially as MMPs, have already been described.

**Methods:** We evaluated IL-1β activation pathways in neutrophils and monocyte subsets from patients with different clinical forms of Chagas disease^1^ after *T. cruzi* antigen stimulation by multiparameter flow cytometry.

**Results:** Our data demonstrated that Chagas patients with the indeterminate clinical form (IND) showed increased levels of IL-1β post-stimulation as well as increased expression of MMP-2, NLRP3, and CASP1, which are associated with the classical caspase-1-dependent pathway. Conversely, patients with the cardiac clinical form (CARD) showed increased IL-1β after stimulation associated with MMP-9 and alternative caspase-1-independent pathway.

**Conclusions:** We suggest some distinct molecular mechanisms for production of IL-1β in innate immune cells from patients with different clinical forms of Chagas disease. MMP-2 and MMP-9 gelatinases are associated with distinct disease outcomes and IL-1β production.

## Introduction

Chagas disease, also known as American trypanosomiasis, is a neglected parasitic disease caused by the protozoan *Trypanosoma cruzi* (1), that affects millions of people in the world (2). During the acute phase, a diffuse and intense inflammation in the cardiomyocytes is observed, which is composed mainly of neutrophils, monocytes, and T lymphocytes. Patients in the chronic phase of Chagas disease are classified in different clinical forms according to the symptoms presented: (1) Indeterminate (IND) with no alterations due the infection (3), (2) cardiac (CARD) with chronic alterations in heart function and structure (4, 5), and (3) digestive with gastrointestinal alterations (6).

Dilated cardiomyopathy is the most severe alteration, affecting about 30% of patients in the chronic phase of the disease (7–9). Chronic chagasic cardiomyopathy is the main fibrosing myocarditis among the known heart diseases (10), with chronic low intensity inflammation, progressive tissue destruction, cardiac remodeling, and extensive fibrosis in the heart (11). The collagen in cardiac matrix is replaced by weak fibers trough matrix metalloproteinases (MMPs), that cleaves extracellular matrix (ECM) proteins (12). MMP-2 and MMP-9 gelatinases received increased attention in cardiac remodeling due to their capacity to degradate more abundant substrates in the heart ECM (12–14). Beyond fibrosis, the gelatinases have been highlighted in some studies for their participation in the immune response, activating cytokines and chemokines, mainly interleukin (IL)-1β, in the central axis of inflammation and regulation (15–17).

IL-1β is a polypeptide of the IL-1 cytokine family, produced by several cell types, specially innate immune cells such as monocytes/macrophages, neutrophils and dendritic cells (18). Among all members of the IL-1 family, IL-1β has been extensively studied for its role in memory responses of innate immunity, fibrosis, inflammation, and heart diseases (18, 19). Production of functional IL-1β requires two steps. First, the transcription of the inactive precursor (pro-IL-1β) is stimulated by a pathogen-associated molecular pattern (PAMP) or a damage-associated molecular pattern (DAMP), but cytokines also may stimulate IL1B gene transcription (19). Classically, the second step occurs when pro-IL-1β is converted to biologically active IL-1β through a process mediated by a caspase-1-dependent pathway, associated with inflammasomes (19).

Evidence points to caspase-1-independent pathways for IL-1β activation (15, 20–23). Given that MMPs role in cardiac remodeling, especially in Chagas' heart disease, involves the synergism between MMP-2 and MMP-9 in fibrosis and the antagonism amongst them in inflammation (13), we investigate their contribution as possible caspase-1-independent pathway for IL-1β activation in *T. cruzi* immunity.

## Population and Methods

### Study Population

The patients who agreed to participate in this study were identified and selected at the Alda Falcão Referral Outpatient Center for Chagas Disease at the *Instituto René Rachou —Fundação Oswaldo Cruz* (IRR/FIOCRUZ), Belo Horizonte, Brazil. Serology for Chagas disease was determined by two or more tests (indirect immunofluorescence, ELISA or indirect hemagglutination) and patients were considered infected when at least two different tests were positive. The patients infected with *T. cruzi* were grouped as indeterminate (IND, *n* = 10) and cardiac (CARD, *n* = 10) patients as previously reported (4, 5). The IND group included asymptomatic individuals ranging in age from 35 to 65 years old (average age, 42 ± 16 years), with no significant alterations in electrocardiography, chest x-ray, echocardiogram, esophagogram, and barium enema. All CARD patients, ranging in age from 35 to 70 years (average age, 51 ± 7 years), presented dilated cardiomyopathy, characterized by the echocardiographic finding of a dilated left ventricle with impaired ventricular systolic function, which were classified as belonging to the group CARD V, as previously reported (5). Left ventricular end-diastolic diameter/body surface area ≥31 mm (average 58 ± 9 mm) and left ventricular ejection fraction <55% (average 36 ± 9%) were used as echocardiographic parameters of Chagas dilated cardiomyopathy. Normal healthy individuals, ranging in age from 35 to 55 years (average age, 44 ± 6 years) and showing negative serological tests for the infection, were from a non-endemic area for Chagas disease and were included as a control group [non-infected (NI, *n* = 6)].

### Ethics Statement

Written informed consent was obtained from all individuals prior to their inclusion in the study. All participants were adults, independent of their participation in this study, all individuals enrolled were submitted to a standard screening protocol, followed up and received clinical treatment. This study was carried out in full accordance with all International and Brazilian accepted guidelines and was approved by the Ethics Committee at IRR/FIOCRUZ/MG (CEPSH-IRR #15/2011).

### *T. cruzi* Soluble Antigen Preparations (TRYPO)

Tissue culture-derived trypomastigotes of *T. cruzi* (CL-Brener strain) was used for TRYPO antigen production. Briefly, parasites were subjected to rupture and homogenization in cold phosphate-buffered saline (PBS), using a glass homogenizer and Teflon pestle, on ice, to prevent overheating. Subsequently, the suspensions were centrifuged at 23.000 g for 60 min at 4°C. The supernatant was collected, dialyzed for 24 h at 4°C against PBS, and sterilized by filtration on 0.22 μm-pore-size membranes. The protein concentration was measured and the material was separated into aliquots and stored at −70°C until use.

### Whole Blood Cultures

A 5 mL of peripheral blood was collected by venipuncture from each subject using a sterile Vacutainer tube containing sodic heparin as anticoagulant. Aliquots of 1 mL of whole blood were mixed with TRYPO diluted in RPMI-1640 medium at final concentration of 20 μg/mL. Aliquots of 1 mL of whole blood were mixed with 1 mL of RPMI-1640 medium for culture control. The samples were incubated for 2 h in CO_2_ incubator with 5% humidity at 37°C, followed by addition of Brefeldin A (1 mg/mL). The incubation continued for 4 h under the same conditions, yielding a total of 6 h of incubation. After incubation, 200 μL of EDTA at a final concentration of 2mM were added directly to the cultures and the samples incubated for 15 min, followed by washing with PBS.

### Flow Cytometry

To the whole blood culture tubes 3 mL were added phosphate-buffered saline to wash (PBS-W, 0.5% BSA, and 0.1% sodium azide), and centrifuged at 400 g for 10 min at 20°C. The supernatant was aspirated leaving a final volume of 2 mL. One hundred microliters of aliquots were mixed in tubes with 2 μL of undiluted monoclonal antibodies anti- CD14 (clone MΦP9) conjugated with peridinin chlorophyll protein complex (PerCP), CD16 (clone 3G8) conjugated with APC-Cy7 (BD Pharmingen™, USA), HLA-ABC (clone W6/32), HLA-DR (clone G46-6), TLR-2 (clone TL2.1), TLR-4 (clone HTA125), CD80 (clone 2D10) and CD86 (clone 2331), CD62L (DREG-56) and CD11b (ICRF44), conjugated with PE-Cy7, FITC, APC, or BV421. After erythrocyte lysis, the cells were washed, permeabilized and incubated with monoclonal antibodies against MMP-2 (clone 1A10), MMP-9 (clone 56129), NLRP3 (clone 768319), IL-1β (clone 8516), IL-10 (clone 127107), IL-12 (clone C11.5), IL-13 (clone JES10), IL-17 (clone BL168), IL-18 (clone 74801), IL-33 (clone 390412), IL-8 (clone E8N1), TNF (clone MAb11), TGF-β (clone TW4-9E7), TLR-9 (eB72-1665), and CASP1 (clone D-3) (Santa Cruz Biotechnologies, R&D Systems, BD Bioscience or Biolegend, USA) conjugated with distinct fluorescence. After incubation, the cells were fixed, and phenotypic analyses performed by flow cytometry using LSR Fortessa™ cytometer (BD Biosciences, USA).

### Acquisition and Analysis Strategy

**Figure 1:**
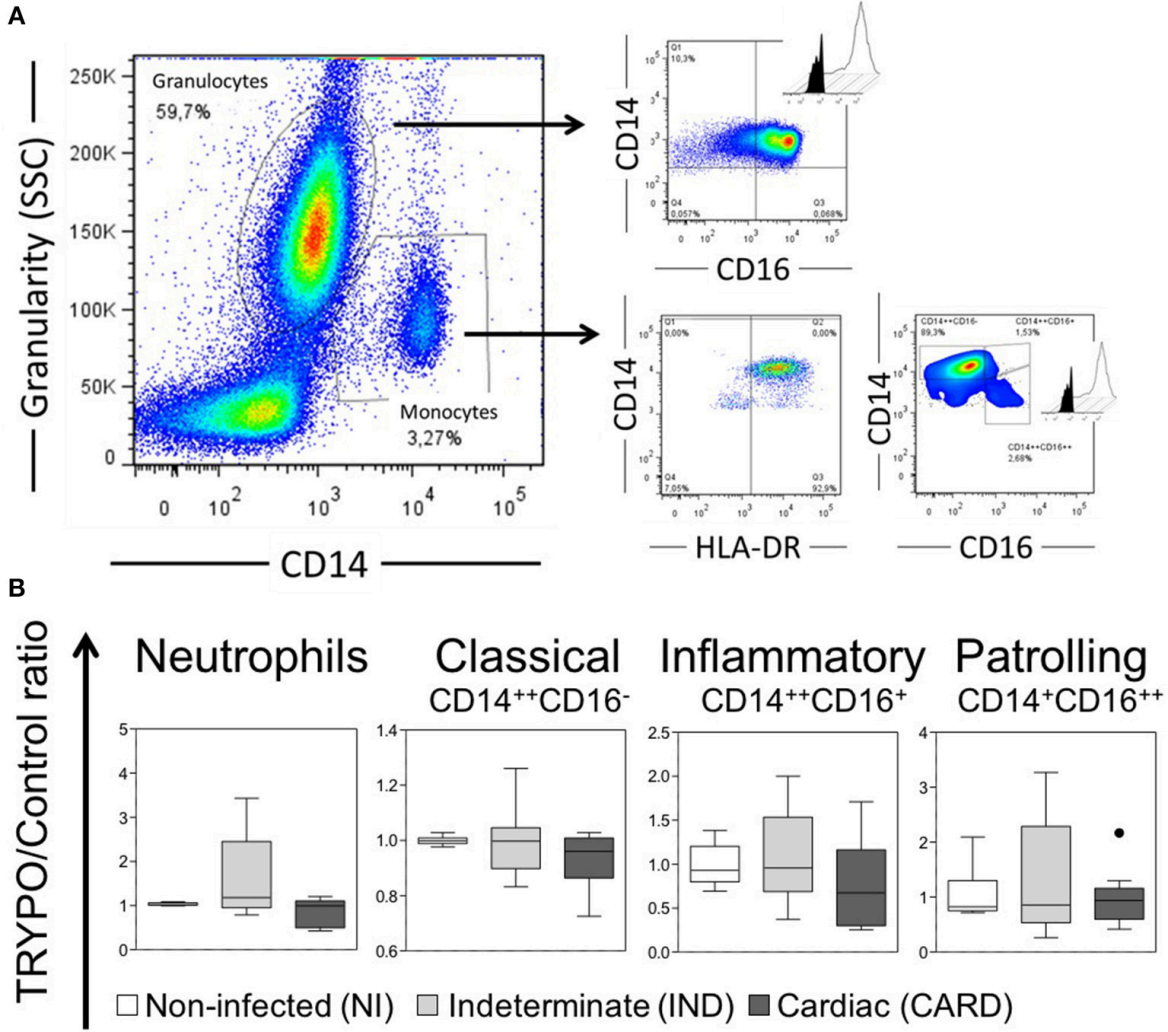
Evaluation of innate immune cells. **(A)** Representative gates of flow cytometry analysis. Histograms show the intracellular expression of the markers evaluated in the medium culture (black) vs. stimulated culture with *T. cruzi* antigens (TRYPO, white). **(B)** Frequency of neutrophils and monocyte subsets in all evaluated groups. Graphs show the frequency of cells ratio of TRYPO vs. medium control cultures in cells from peripheral blood of non-infected individuals (NI, *n* = 06), patients with indeterminate (IND, *n* = 10) and cardiac (CARD, *n* = 10) clinical forms of Chagas disease. Boxes show the median and interquartile ranges, whiskers indicate highest and lowest observations and dots represent the outliers.

A total of 7 × 10^4^ events were acquired/sample, and granulocytes and total monocytes were gated based on anti-CD14 vs. side scatter (SSC) plot (Figure 1A). All data were collected and analyzed using FlowJo software. Neutrophils analysis was performed using a dot plot of intermediate CD14 and high CD16 expression (Figure 1A). Monocytes were selected from positive expression of CD14 and HLA-DR, and monocyte subsets classified in classical CD14^++^CD16^−^, inflammatory CD14^++^CD16^+^, and patrolling CD14^+^CD16^++^ (Figure 1A). The intracellular expression of molecules in monocytes and neutrophils were mensured by mean fluorescence intensity (MFI) or frequency (%) of cytokines^+^ cells in histogram.

### Statistical Analysis

Statistical analyses were performed using GraphPad Prism 5.0 software package (San Diego, USA). All data files assume a non-Gaussian distribution and statistical comparisons were carried out using the non-parametric Kruskal–Wallis test, followed by Dunn's multiple comparison test for NI, IND and CARD groups. Correlation analysis was done using Spearman's correlation coefficient by JMP software (Cary, NC, USA). Principal component analyses (PCA) were performed using the ClustVis web tool as described by Metsalu and Vilo (24). In all cases, significance was considered at *p* < 0.05.

## Results

### Neutrophils From IND and CARD Are Less Active Than NI

**Figure 2:**
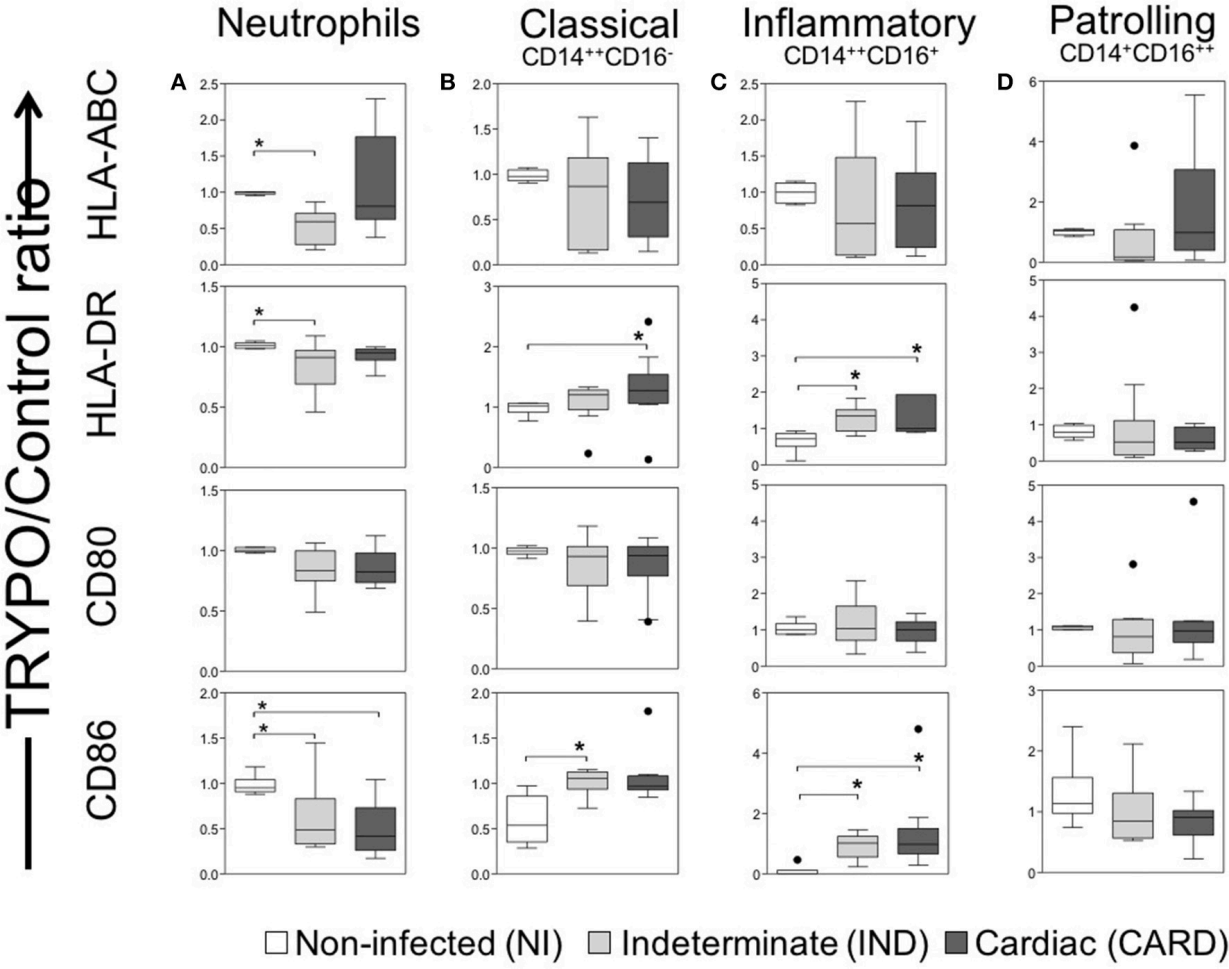
Evaluation of activation and co-stimulation molecules in innate immune cells. Expression of activation molecules HLA-DR and HLA-ABC and co-stimulatory molecules CD80 and CD86 were evaluated in neutrophils **(A)**, classical CD14^+^CD16^−^ monocytes **(B)**, inflammatory CD14^++^CD16^+^ monocytes **(C)** and patrolling CD14^+^CD16^−^ monocytes **(D)**. Graphs show the mean intensity of fluorescence (MFI) ratio of TRYPO vs. medium control cultures in cells from peripheral blood of non-infected individuals (NI, *n* = 6), patients with indeterminate (IND, *n* = 10) and cardiac (CARD, *n* = 10) clinical forms of Chagas disease. Statistical differences (*p* < 0.05) between groups were obtained by Kruskal-Wallis test, followed by Dunn's post-test, and showed by asterisk (^*^) and lines. Boxes show the median and interquartile ranges, whiskers indicate highest and lowest observations and dots represent the outliers.

The percentage of neutrophils was similar between NI, IND and CARD groups (Figure 1B). Neutrophils from IND group showed lower expression of HLA-ABC, HLA-DR and CD86 as compared to NI (Figure 2A and Supplementary Figure 1). Neutrophils from CARD group also showed a decrease in CD86 expression than NI group (Figure 2A and Supplementary Figure 1). We observed no difference in CD80 expression by neutrophils between groups (Figure 2A and Supplementary Figure 1).

### Classical and Inflammatory Monocytes Showed High CD86 Expression

The percentage of monocyte subsets was similar between NI, IND, and CARD groups (Figure 1B). Our data did not show any differences in HLA-ABC and CD80 expression by all monocyte subsets between NI, IND, and CARD groups (Figures 2B–D and Supplementary Figure 1). Classical and inflammatory monocytes from CARD group showed higher expression of HLA-DR than NI (Figures 2B,C and Supplementary Figure 1). There was no difference between the IND and NI groups on HLA-DR expression (Figures 2B,C). Classical monocytes from IND group had higher CD86 expression compared to NI group (Figure 2B and Supplementary Figure 1). There was no difference between the CARD group and the NI group regarding the expression of CD86 (Figure 2B). Patrolling monocytes showed no variation in any activation and co-stimulatory molecules between NI, IND, and CARD groups (Figure 2D).

### Neutrophils and Monocytes From IND and CARD Expressed Higher Level of IL-1β

**Figure 3:**
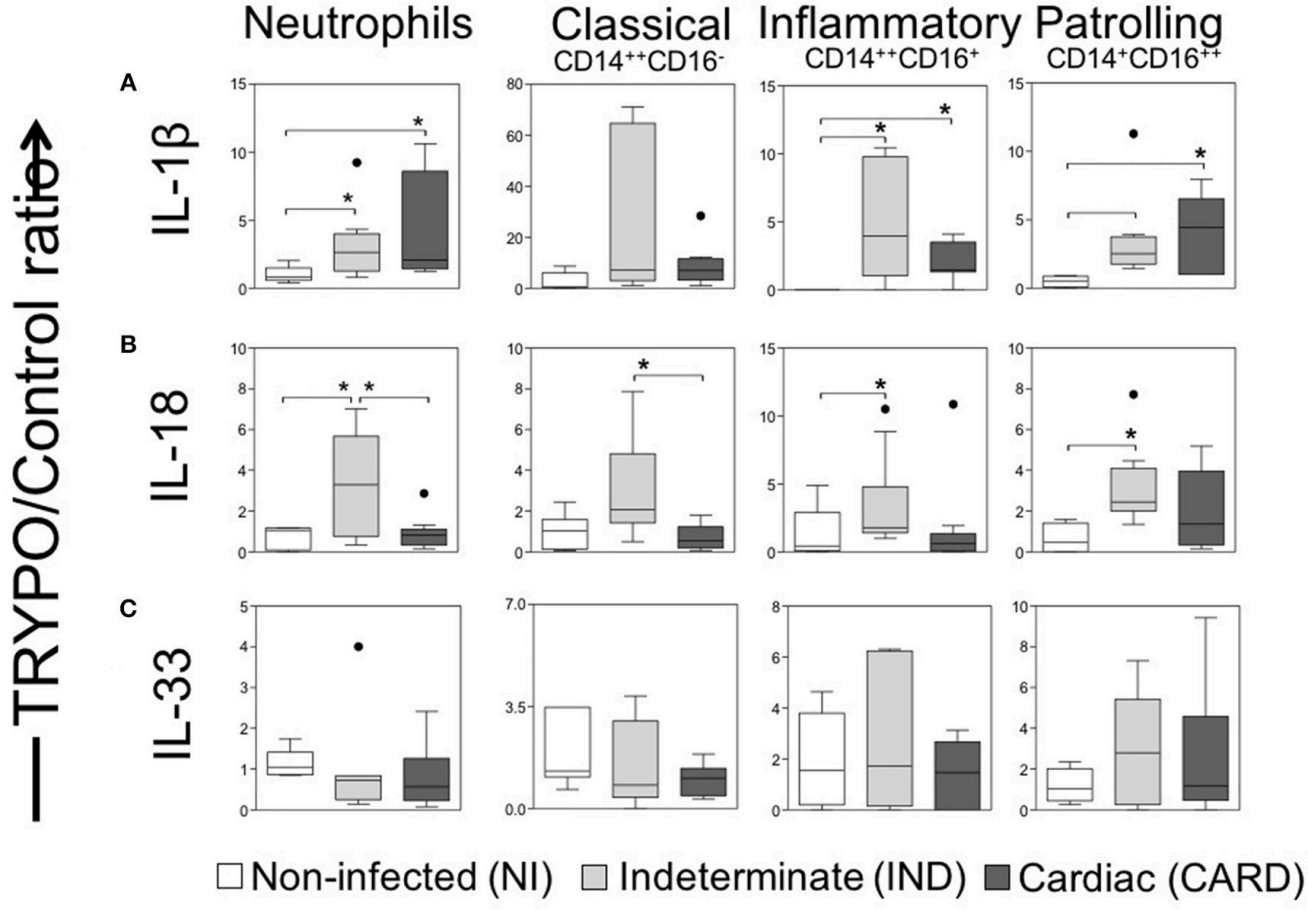
Evaluation of IL-1 family cytokines in innate immune cells. Expression of IL-1β **(A)**, IL-18 **(B)** and IL-33 **(C)** were evaluated in neutrophils, classical CD14^+^CD16^−^ monocytes, inflammatory CD14^++^CD16^+^ monocytes and patrolling CD14^+^CD16^−^ monocytes. Graphs show the frequency of cytokine^+^ cell ratio of TRYPO vs. medium control cultures in cells from peripheral blood of non-infected individuals (NI, *n* = 06), patients with indeterminate (IND, *n* = 10) and cardiac (CARD, *n* = 10) clinical forms of Chagas disease. Statistical differences (*p* < 0.05) between groups were obtained by Kruskal-Wallis test, followed Dunn's post-test, and showed by asterisk (^*^) and lines. Boxes show the median and interquartile ranges, whiskers indicate highest and lowest observations and dots represent the outliers.

We observed higher frequency of neutrophils, inflammatory and patrolling monocytes expressing IL-1β in IND and CARD groups as compared to NI (Figure 3A).

Our data demonstrated a higher frequency of neutrophils from IND positive to IL-18 as compared to NI, as well as compared to CARD (Figure 3B). We also observed a higher frequency of inflammatory and patrolling monocytes expressing IL-18 in IND than NI group, while classical monocytes positives for IL-18 were increased in IND compared to CARD (Figure 3B). The frequency of positive cells for IL-33 was similar for all cells among the different groups (Figure 3C).

### TRYPO Recognition Seems to be by TLR-4 in IND and CARD

**Figure 4:**
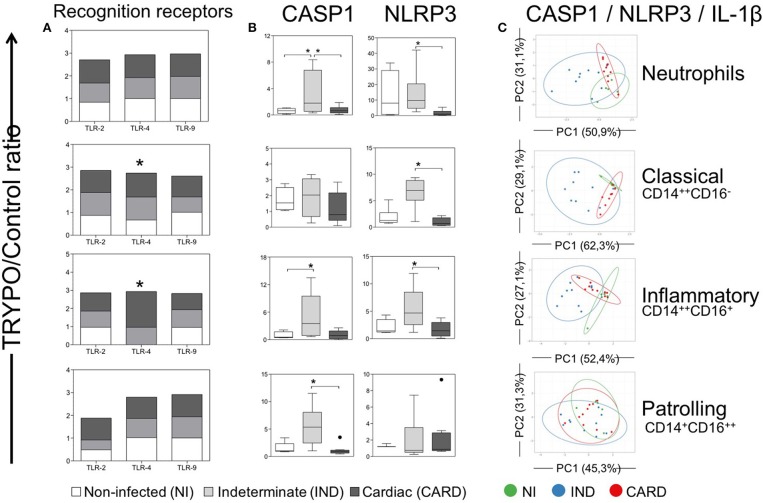
Evaluation of classical caspase-1-dependent pathway in innate immune cells. Expression of toll-like receptors (TLR)−2,−4, and−9 **(A)**, CASP1 and NLRP3 **(B)** and principal component (PC) analysis between CASP1, NLRP3, and IL-1β **(C)** were evaluated in neutrophils, classical CD14^+^CD16^−^ monocytes, inflammatory CD14^++^CD16^+^ monocytes and patrolling CD14^+^CD16^−^ monocytes. Graphs show the mean intensity of fluorescence (MFI) ratio of TRYPO vs. medium control cultures in cells from peripheral blood of non-infected individuals (NI, *n* = 6), patients with indeterminate (IND, *n* = 10) and cardiac (CARD, *n* = 10) clinical forms of Chagas disease. Statistical differences (p < 0.05) between groups were obtained by Kruskal-Wallis test, followed by Dunn's post-test, and showed by asterisk (*) and/or lines. Boxes show the median and interquartile ranges, whiskers indicate highest and lowest observations and dots represent the outliers.

Our data demonstrated that TLR-2 and TLR-9 expression were equivalent in all cells between NI, IND and CARD groups after *in vitro* TRYPO stimulation (Figure 4A). TLR-4 expression was higher in classical and inflammatory monocytes from IND and CARD compared to NI (Figure 4A). Neutrophils and patrolling monocytes showed no differences in TLR-4 expression between NI, IND, and CARD groups (Figure 4A).

### IND Showed Higher Expression of Caspase-1-Dependent IL-1β Activation Pathway Molecules

We evaluated NLRP3 and CASP1 expression, which are molecules related to the classical caspase-1-dependent IL-1β activation pathway. Our data showed that neutrophils from IND group present higher CASP1 expression compared to the NI and CARD groups (Figure 4B). However, the inflammatory monocytes have increased CASP1 expression in IND compared to NI group, while patrolling monocytes showed higher CASP1 expression compared only to the CARD group (Figure 4B). We have also observed that neutrophils, classical and inflammatory monocytes from IND showed higher expression of NLRP3 than NI and CARD groups (b).

PCA is a method in which a multivariate data set is linearly transformed into a set of uncorrelated variables, sorted by the variance. In this way, we can interpret the first principal components (PC) accounts for as much of the variability in the data as possible, and each succeeding PC accounts for as much of the remaining variability. Thus, it is possible to verify if some of the sample groups overlap or form separated clusters. Our PCA demonstrated that classical caspase-1-dependent IL-1β activation pathway cluster separately and therefore are considered good molecular markers to segregate individuals from the different clinical forms (Figure 4C).

### Distinct Cytokines Profile Induced by TRYPO Stimulation in IND and CARD

**Figure 5:**
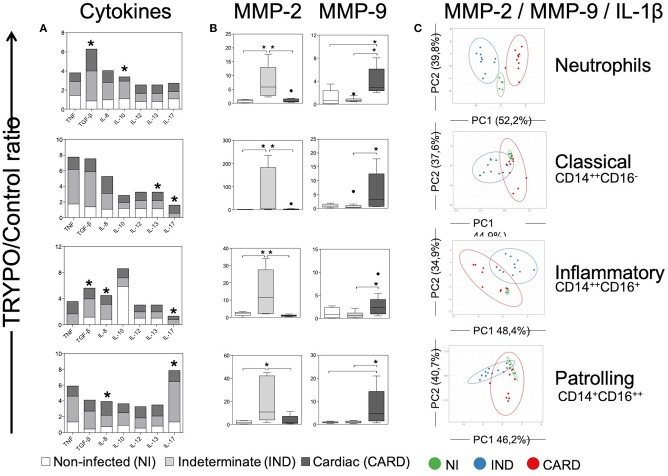
Evaluation of alternative caspase-1-independent pathway in innate immune cells. Expression of cytokines **(A)**, MMP-2 and MMP-9 **(B)** and principal component (PC) analysis between MMP-2, MMP-9, and IL-1β **(C)** were evaluated in neutrophils, classical CD14^+^CD16^−^ monocytes, inflammatory CD14^++^CD16^+^ monocytes and patrolling CD14^+^CD16^−^ monocytes. Graphs show the mean intensity of fluorescence (MFI) or the frequency of cytokine^+^ cells ratio of TRYPO vs. medium control cultures in cells from peripheral blood of non-infected individuals (NI, *n* = 6), patients with indeterminate (IND, *n* = 10) and cardiac (CARD, *n* = 10) clinical forms of Chagas disease. Statistical differences (*p* < 0.05) between groups were obtained by Kruskal-Wallis test, followed by Dunn's post-test, and showed by asterisk (^*^) and/or lines. Boxes show the median and interquartile ranges, whiskers indicate highest and lowest observations and dots represent the outliers.

Neutrophils from IND group showed higher TGF-β and IL-10 expression than NI and CARD groups (Figure 5A). No difference was observed in TNF, IL-8, IL-12, IL-13, and IL-17 expression by neutrophils between NI, IND and CARD groups (Figure 5A).

Classical monocytes from IND and CARD showed high expression of IL-13 and IL-17 compared to NI group (Figure 5A). No difference was observed in TNF, TGF-β, IL-8, IL-10, and IL-12 expression by classical monocytes between NI, IND and CARD groups (Figure 5A).

We also demonstrated that inflammatory monocytes from IND and CARD a higher expression of TGF-β, IL-8, and IL-17 compared to NI group (Figure 5A). TNF, IL-10, IL-12, and IL-13 expression was similar in the same cells between groups evaluated (Figure 5A).

Patrolling monocytes from IND group expressed high level of IL-17, while cells from CARD group expressed high levels of IL-8, both compared to NI groups (Figure 5A). TNF, TGF-β, IL-10, IL-12, and IL-13 expression was similar in patrolling monocytes between evaluated groups (Figure 5A).

### CARD Show Higher Expression of Caspase-1-Independent IL-1β Activation Pathway Molecules

We evaluated MMP-2 and MMP-9 expression, which are enzymes that may cleave pro-IL-1β in activated fragment of IL-1β according to an alternative caspase-1-independent IL-1β activation pathway. Our data show that neutrophils and all monocyte subsets from IND expressed higher levels of MMP-2 as compared to NI and CARD groups (Figure 5B). In contrast, all cells from CARD expressed higher levels of MMP-9 than NI and IND groups (Figure 5B).

PCA analysis demonstrated that alternative caspase-1-independent IL-1β activation pathway cluster separately and therefore are considered good molecular markers to segregate individuals with the different clinical forms, mainly in the neutrophils population (Figure 5C).

### MMP-2 and MMP-9 Are Differently Associated With IL-1β Activation Pathways

Neutrophils and classical monocytes showed a positive correlation of MMP-2 with IL-1β, CASP1, and NLRP3 (Table 1). These cells also demonstrated a positive correlation between CASP1 and NLRP3 (Table 1). On the other hand, MMP-9 showed a negative correlation with CASP1 and NLRP3 (Table 1).

**Table 1:** Correlations analysis for IL-1β activation pathways.

**Variable**	**by variable**	***R*^**2**^**	***p*-value**	**Population**	**Variable**	**by variable**	***R*^**2**^**	***p*-value**	**Population**
MMP-9	MMP-2	−0.2274	0.2638	Neutrophils CD16^++^CD14^+^	MMP-9	MMP-2	−0.3388	0.0905	Inflammatory Monocytes CD14^++^CD16^+^
IL-1β	MMP-2	0.3988	0.0436^*^		IL-1β	MMP-2	0.1083	0.5986	
IL-1β	MMP-9	0.2565	0.2059		IL-1β	MMP-9	0.2458	0.2262	
CASP1	MMP-2	0.4818	0.0127^*^		CASP1	MMP-2	0.3203	0.1106	
CASP1	MMP-9	−0.6344	0.0443^*^		CASP1	MMP-9	−0.2167	0.2875	
CASP1	IL-1β	0.24	0.2375		CASP1	IL-1β	0.2189	0.2826	
NLRP3	MMP-2	0.7093	<.0001^*^		NLRP3	MMP-2	0.6052	0.0011^*^	
NLRP3	MMP-9	−0.5144	0.0072^*^		NLRP3	MMP-9	−0.2699	0.1823	
NLRP3	IL-1β	0.0858	0.6767		NLRP3	IL-1β	0.3504	0.0793	
NLRP3	CASP1	0.4715	0.015^*^		NLRP3	CASP1	0.2758	0.1727	
MMP-9	MMP-2	−0.0462	0.8228	Classical Monocytes CD14^++^CD16^−^	MMP-9	MMP-2	−0.0359	0.8617	Patrolling Monocytes CD14^+^CD16^++^
IL-1β	MMP-2	0.3935	0.0467^*^		IL-1β	MMP-2	0.3456	0.0837	
IL-1β	MMP-9	0.1952	0.3392		IL-1β	MMP-9	0.026	0.8997	
CASP1	MMP-2	0.4373	0.0255^*^		CASP1	MMP-2	0.2202	0.2796	
CASP1	MMP-9	−0.6274	0.0006^*^		CASP1	MMP-9	−0.3112	0.1218	
CASP1	IL-1β	−0.0797	0.6989		CASP1	IL-1β	0.1751	0.3923	
NLRP3	MMP-2	0.7005	<0.0001^*^		NLRP3	MMP-2	0.24	0.2377	
NLRP3	MMP-9	−0.4318	0.0276^*^		NLRP3	MMP-9	−0.0306	0.882	
NLRP3	IL-1β	0.3607	0.0703		NLRP3	IL-1β	0.0388	0.8507	
NLRP3	CASP1	0.5487	0.0037^*^		NLRP3	CASP1	−0.0027	0.9894	

*MMP, Matrix metalloproteinase; IL, Interleukin; CASP, Caspase; NLR, NOD-like receptor*.

* *Indicates statistically significant difference (p < 0.05) by Spearman coefficient*.

Inflammatory monocytes showed only a positive correlation between MMP-2 and NLRP3. However, significaticant correlations were not observed in patrolling monocytes (Table 1).

**Figure 6:**
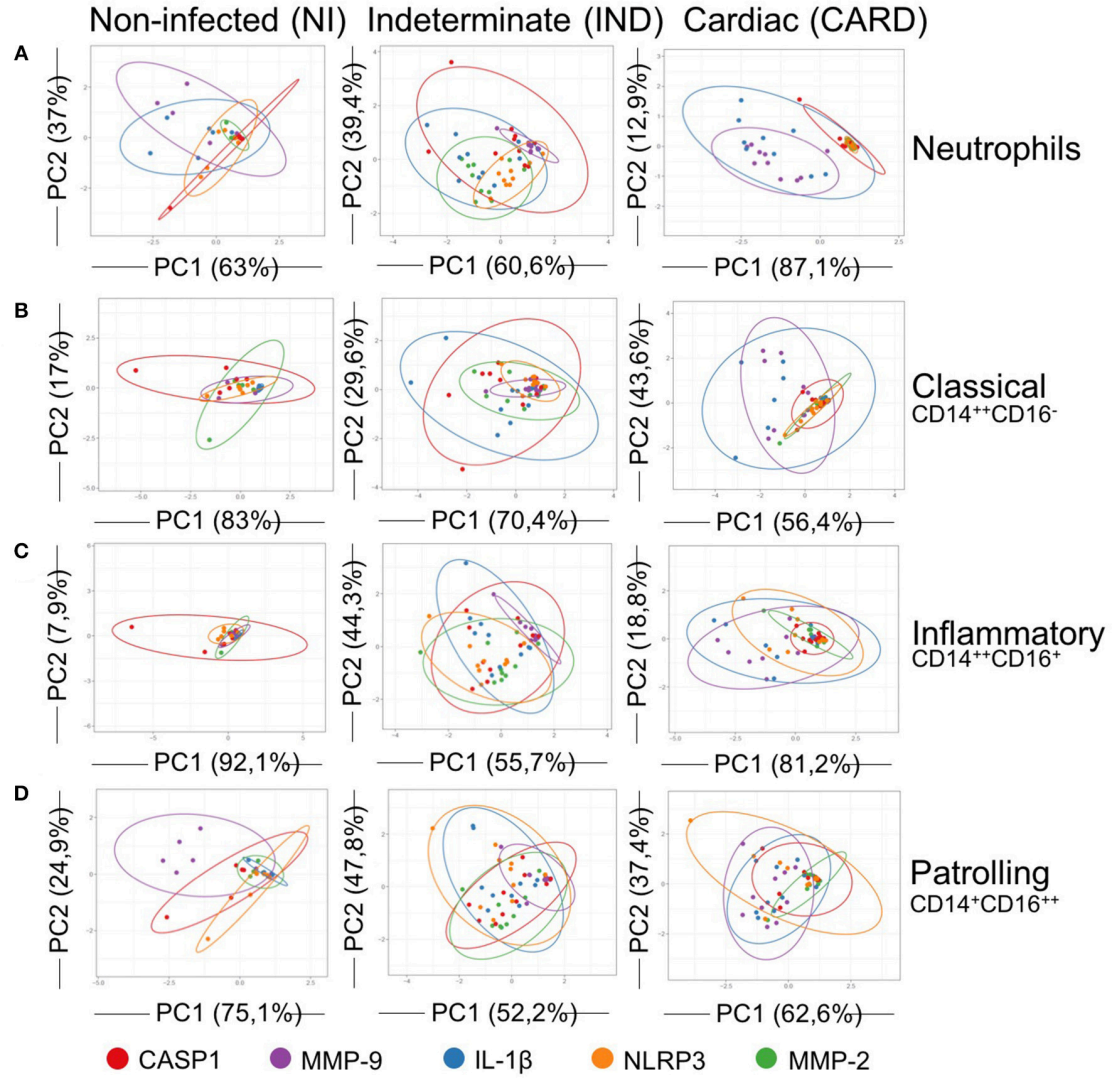
Principal components analysis (PCA) of IL-1β activation pathways. PCA demonstrate a scatter diagram with axes corresponding two different principal components (PC) for expression of CASP1 (red), MMP-9 (purple), IL-1β (blue), NLRP3 (orange) and MMP-2 (green). Neutrophils **(A)**, classical CD14^+^CD16^−^ monocytes **(B)**, inflammatory CD14^++^CD16^+^ monocytes **(C)**, and patrolling CD14^+^CD16^−^ monocytes **(D)** from peripheral blood of non-infected individuals (NI, *n* = 06) and patients with indeterminate (IND, *n* = 10) and cardiac (CARD, *n* = 10) clinical forms of Chagas disease were evaluated. X and Y axes show the PC1 and the PC2, which explain the percentage of total variance, respectively. Prediction ellipses show how groups differ. The confidence level established for the size of the ellipses is 0.95.

IND and CARD showed a different clusterization with IL-1β pathways molecules PCA analysis of each molecule from IL-1β activation pathways demonstrated the occurrence of disorganized clusters, that did not show a clear standard for all cells in NI group (Figure 6).

Neutrophils and classical and inflammatory monocytes from IND showed cluster overlaps between IL-1β and CASP1 (Figures 6A–C). Patrolling monocytes of IND group demonstrated cluster overlap for IL-1β and NLRP3 (Figure 6D). IND group remained the standard IL-1β clustered with molecules of classical caspase-1-dependent pathway.

On the other hand, all cells from CARD showed a standard pattern with overlay cluster between MMP-9 and IL-1β (Figures 6A–D).

## Discussion

Fibrosis and inflammation are the underlying mechanisms of remodeling in Chagas cardiomyopathy. IL-1β is a cytokine that influences both processes, especially by the induction of TGF-β (25). Other important molecules that have received significant attention due to the same dichotomous characteristic are MMPs. We previously described that MMP-2 and MMP-9 gelatinases mechanisms in chronic phase of Chagas disease, where we observed a synergism in fibrosis and an antagonism in inflammation (13).

Here, we confirm that MMP-2 and−9 gelatinases interact contrariwise with inflammation mediated by IL-1β. MMP-2 was highly expressed by IND group cells, showed positive correlation with IL-1β. MMP-2 and also correlated positively with CASP1 and NLRP3, which are molecules of the classical caspase-1-dependent activation pathway for IL-1β (26). On the other hand, MMP-9 is an alternative activation pathway for IL-1β (15, 21, 22), and we demonstrated a negative correlation with CASP1 and NLRP3, and a positive correlation with IL-1β.

When IL-1β secretion is evaluated it is important to consider the stimulus inducing release. The mechanism of secretion may be influenced by the type stimulus and its intensity (27). IL-1β is translated on free polyribosomes associated with the cytoskeleton, and not membrane-bound polyribosomes (28), it is released in response to many PAMPs and DAMPs. Most of the IL-1β produced is located in the cytosol and another small fraction is directed to endolysosomal vesicles, protected from tryptic digestion (29). The vesicular fraction of IL-1β cellular is directed to degradation, however, this segment may be rescued by triggering lysosome exocytosis and thus secretion of IL-1β (30).

Our data suggest that different IL-1β activation pathways are clearly involved in the chronic phase of Chagas disease. However, it has distinct mechanisms. While IND patients seems to produce IL-1β through a classical pathway mediated by MMP-2, NLRP3, and CASP1, CARD patients it is induced by MMP-9 promoting fibrosis and inflammation in the heart by IL-1β activation, among other mechanisms.

Another route that a fraction of cellular IL-1β can leave the cell is via a protected form when it is packaged and secreted via exosomes or microvesicles from the plasma membrane (31). The secretion of these exosomes is dependent upon NLRP3, but independent of CASP1 (32). IL-1β is secreted directly by the cell and has a relatively short half-life in the plasma (33). The exosome increase half-life and range of action of IL-1β, thus it can reach more distant lesions.

Indeed, exosomes secreted by cardiomyocytes subjected to ischemia display higher levels of MMPs and promote their secretion by endothelial cells (34). We suggest that elevated levels of MMP-9 in cells from CARD patients contribute to the cleavage of IL-1β in an active fragment through this mechanism.

IND patients seems to produce IL-1β by classical caspase-1-dependent pathway. Our data showed an association of IL-1β with NLRP3 and CASP1 in this group. IND patients have a well-established regulatory condition by production of anti-inflammatory cytokines, such as IL-10 (35, 36). Here, we also demonstrated this condition in neutrophils. Gurung and colleagues demonstrated that chronic LPS exposure triggers regulatory mechanisms to dampen NLRP3 activation. They identified IL-10 as the secreted inflammasome-tolerizing factor that acts in an autocrine manner to control activation of the NLRP3 inflammasome. These evidence suggest that IL-10 dampen NLRP3 inflammasome activation to avoid excessive inflammation (37). In fact, our group has previously shown that IL-10 may be associated with host protection against the excessive pathology induced by type 1 responses (38). It is possible that IL-10, together with NLRP3, can maintain a balance between parasitism and tissue integrity in IND patients. This may also work to contain the effects of IL-1β under the fibrosis and inflammation in IND patients, avoiding Chagas' heart disease.

In addition to the high IL-1β expression in infected groups, we also evaluated the expression of other IL-1 family cytokine, such as IL-18 and IL-33. IL-18 is a potent inducer of IFN-γ (19, 39), and a mediator of both Th1 and Th2 responses. We demonstrated higher expression of IL-18 in neutrophils from IND group, which corroborate with other studies that also described high levels of IFN-γ in cells of these patients (40). IL-33 functions as an alarm signal released upon cell injury or tissue damage, that emerges as a crucial immune modulator with pleiotropic activities in regulatory immune responses (41). We did not observe differences regarding the expression of IL-33 in our groups.

Chagas cardiomyopathy is an important model to study heart disease due its characteristics of intense fibrosis and inflammation. One of the major challenges in chronic Chagas disease is to understand the mechanism by which clinical intervention is more effective and capable of preventing the clinical evolution from IND to CARD, or to minimize the effects of fibrosis on the heart of those patients with light/moderate heart alterations. Our data propose for the first time a distinct molecular mechanisms for the production of IL-1β in patients with different clinical forms of Chagas disease. IND patients showed IL-1β production associated with higher expression of MMP-2, NLRP3, and CASP1, whereas IL-1β in CARD patients were correlated with MMP-9 and alternative caspase-1-independent pathway. MMP-2 and MMP-9 gelatinases are involved differently in this process, but other molecules, such as IL-10, seem to influence the final consequences of fibrosis and inflammation in Chagas heart disease. Due this dichotomous characteristic in the activation of IL-1β in patients with different clinical forms of Chagas' disease, we propose MMP-2 and MMP-9 as potential biomarkers of prognostic which should be better investigated. Likewise, the different pathways of IL-1β activation demonstrated here may be studied as a parameter of pharmacological intervention in order to prevent the evolution of cardiomyopathy, fibrosis and inflammation in the pathogenesis of Chagas disease.

## Ethics Statement

This study was carried out in full accordance with all International and Brazilian accepted guidelines and was approved by the Ethics Committee at IRR/FIOCRUZ/MG (CEPSH-IRR #15/2011).

## Author Contributions

WD, RC-O, and JG contributed conception and design of the study. NM and BP performed the experiments. NM and LM organized the database and performed the statistical analysis. NM wrote the first draft of the manuscript. AT-C and SE-S select and lead clinical management of patients. All authors contributed to manuscript revision, read, and approved the submitted version.

### Conflict of Interest Statement

The authors declare that the research was conducted in the absence of any commercial or financial relationships that could be construed as a potential conflict of interest. The handling Editor declared a past collaboration with one of the authors WD.

## Supplementary Material

Supplementary Figure 1 Evaluation of activation and co-stimulation molecules in innate immune cells. Expression of HLD-ABC **(A)**, HLA-DR **(B)**, CD80 **(C)**, and CD86 **(D)** were measured as mean fluorescence intensity (IMF) in neutrophils, classical CD14^+^CD16^−^ monocytes, inflammatory CD14^++^CD16^+^ monocytes and patrolling CD14^+^CD16^−^ monocytes of peripheral blood from non-infected individuals (NI, *n* = 6) and patients with indeterminate clinical forms (IND, *n* = 10) and cardiac (CARD, *n* = 10) of Chagas disease. The bars show the median and the interquartile range, the vertical lines indicate the lower and upper limits, while the points represent the outliers. Significant differences (*p* < 0.05) between control without stimulus (bars without filling) and culture stimulated with T. cruzi antigens (bars filled with diagonal lines) in the same group were identified according to the Wilcoxon paired test and by asterisks (^*^) and lines. Significant differences between the evaluated groups were calculated by the Kruskal–Wallis test and represented by the letters a (control without stimulus—Medium) and b (after stimulation—TRYPO).
