# Supplementary figures and images for: Evidence of Different IL-1β Activation Pathways in Innate Immune Cells From Indeterminate and Cardiac Patients With Chronic Chagas Disease
Source: Front Immunol. 2019 Apr 18;10:800. doi: 10.3389/fimmu.2019.00800 (PMC6482163; doi:10.3389/fimmu.2019.00800)

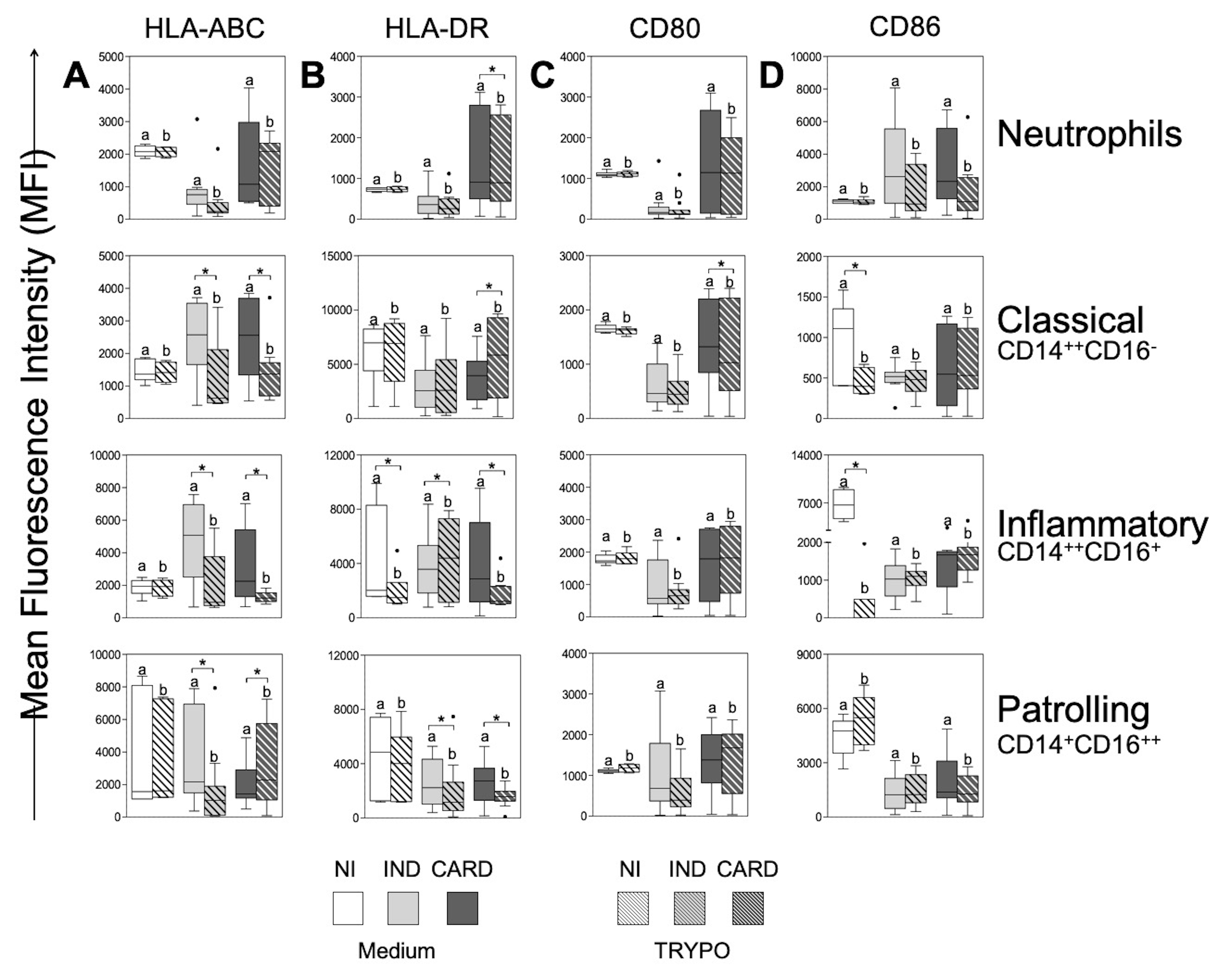

Supplement: Supplementary Figure 1 — Evaluation of activation and co-stimulation molecules in innate immune cells. Expression of HLD-ABC (A), HLA-DR (B), CD80 (C), and CD86 (D) were measured as mean fluorescence intensity (IMF) in neutrophils, classical CD14+CD16− monocytes, inflammatory CD14++CD16+ monocytes and patrolling CD14+CD16− monocytes of peripheral blood from non-infected individuals (NI, n = 6) and patients with indeterminate clinical forms (IND, n = 10) and cardiac (CARD, n = 10) of Chagas disease. The bars show the median and the interquartile range, the vertical lines indicate the lower and upper limits, while the points represent the outliers. Significant differences (p < 0.05) between control without stimulus (bars without filling) and culture stimulated with T. cruzi antigens (bars filled with diagonal lines) in the same group were identified according to the Wilcoxon paired test and by asterisks (*) and lines. Significant differences between the evaluated groups were calculated by the Kruskal–Wallis test and represented by the letters a (control without stimulus—Medium) and b (after stimulation—TRYPO). [file Image_1.TIFF]
